# Supplementary material for: Patterns of Perceived Indoor Environment in Danish Homes
Source: Int J Environ Res Public Health. 2022 Sep 13;19(18):11498. doi: 10.3390/ijerph191811498 (PMC9517311; doi:10.3390/ijerph191811498)
Supplement: Supplementary file 1 [file ijerph-19-11498-s001.zip › ijerph-1897148-supplementary.pdf]

Patterns of perceived indoor environment in Danish homes

Supplementary Materials.

**Table S1**	Questions used to assess perceived indoor environment

Table S1. Questions used to assess perceived indoor environment

**Question***	**Response options**
Have you, in your residence, been annoyed by any of the following within the last 14 days?<br><br>No, not annoyed by any of the following<br><br>a) Too high or low temperatures<br>b) Draught<br>c) Draught along the floor<br>d) Stuffy air<br>e) Poor drinking water<br>f) Noise from traffic<br>g) Noise from installations (e.g., drain, radiator, refrigerator)<br>h) Noise from neighbors<br>i) Noise from nearby industry<br>j) Infrasound or low frequency sound<br>k) Vibrations in building (from e.g., traffic)<br>l) Static electricity<br>m) Too little light<br>n) Skin irritation after use of hot water	Yes, very annoyed<br>yes, slightly annoyed<br>no, not annoyed
Was the residence placed next to a road with through traffic?	Yes<br>No

*Questions have been translated from Danish
